# The prevalence of disrespect and abuse during facility-based childbirth in urban Tanzania

**DOI:** 10.1186/s12884-016-1019-4

**Published:** 2016-08-19

**Authors:** David Sando, Hannah Ratcliffe, Kathleen McDonald, Donna Spiegelman, Goodluck Lyatuu, Mary Mwanyika-Sando, Faida Emil, Mary Nell Wegner, Guerino Chalamilla, Ana Langer

**Affiliations:** 1 Management and Development for Health (MDH), P O Box 79804, Dar es Salaam, Tanzania; 2 Women and Health Initiative, Department of Global Health and Population, Harvard School of Public Health, Boston, USA; 3 Department of Epidemiology and Department of Biostatistics, Harvard School of Public Health, Boston, USA; 4 African Academy of Public Health (APH), Dar es Salaam, Tanzania; 5 Department of Global Health and Population, Harvard School of Public Health, Boston, USA; 6 Ariadne Labs at Brigham and Women’s Hospital and the Harvard T.H. Chan School of Public Health, Boston, Massachusetts USA; 7 Boston University School of Public Health, Boston, USA

**Keywords:** Disrespect, Abuse, Respectful maternity care, Facility delivery, Maternal health, Tanzania

## Abstract

**Background:**

In many countries, rates of facility-based childbirth have increased substantially in recent years. However, insufficient attention has been paid to the acceptability and quality of maternal health services provided at facilities and, consequently, maternal health outcomes have not improved as expected. Disrespect and abuse during childbirth is increasingly being recognized as an indicator of overall poor quality of care and as a key barrier to achieving improved maternal health outcomes, but little evidence exists to describe the scope and magnitude of this problem, particularly in urban areas in low-income countries.

**Methods:**

This paper presents findings from an assessment of the prevalence of disrespectful and abusive behaviors during facility-based childbirth in one large referral hospital in Dar es Salaam, Tanzania. Client reports of disrespect and abuse (D&A) were obtained through postpartum interviews immediately before discharge from the facility with 1914 systematically sampled women and from community follow-up interviews with 64 women four to six weeks post-delivery. Additionally, 197 direct observations of the labor, delivery, and postpartum period were conducted to document specific incidences of disrespect and abuse during labor and delivery, which we compared with women’s reports.

**Results:**

During postpartum interviews, 15 % of women reported experiencing at least one instance of D&A. This number was dramatically higher during community follow-up interviews, in which 70 % of women reported any experience of D&A. During postpartum interviews, the most common forms of D&A reported were abandonment (8 %), non-dignified care (6 %), and physical abuse (5 %), while reporting for all categories of D&A, excluding detention and non consented care, was above 50 % during community follow-up interviews. Evidence from direct observations of client-provider interactions during labor and delivery confirmed high rates of some disrespectful and abusive behaviors.

**Conclusions:**

This study is one of the first to quantify the prevalence of disrespect and abuse during facility-based childbirth in a large public hospital in an urban setting. The difference in respondent reports between the two time periods is striking, and more research is needed to determine the most appropriate methodologies for measuring this phenomenon. The levels and types of disrespect and abuse reported here represent fundamental violations of women’s human rights and are symptomatic of failing health systems. Action is urgently needed to ensure acceptable, quality, and dignified care for all women.

## Background

The advent of the Millennium Development Goals (MDG) in 2000 compelled national and international governing bodies to prioritize the reduction of maternal mortality. The leading causes of maternal death worldwide—hemorrhage, hypertensive disorders, complications of unsafe abortions, and sepsis—can be either prevented or treated in most cases, and the vast majority of deaths from these causes could be eliminated if women were afforded timely care by skilled health professionals at a well-equipped facility [1]. Universal facility-based delivery has, therefore, been widely promoted as an effective means of reducing maternal mortality.

After the adoption of the MDGs, efforts to promote facility-based delivery have focused primarily on increasing access to childbirth services. This focus on access has been documented in Tanzania, where there has been particular emphasis on increasing the number of facilities equipped to provide safe delivery and where accessibility has been improved through the elimination of user fees for maternal health services [2]. However, mounting evidence shows that increasing facility-based delivery rates alone is insufficient to reduce maternal mortality [3]. A higher standard of quality for maternal health services, which comprehensively addresses the physical health and overall wellbeing of clients, is imperative for ensuring impact on maternal health outcomes [4]. Unfortunately, insufficient attention has been paid to the quality and acceptability of services provided to women in the facilities at which they are being encouraged to deliver [5]. Thus, while basic coverage of maternal health services has increased in Tanzania, progress towards reducing maternal mortality has lagged behind [6]. This is also the case in many urban areas throughout other resource limited countries, where, due in no small part to poor quality care, increased rates of facility-based deliveries have not had the expected commensurate impact on lowering maternal mortality [5]. As the importance of quality of care is progressively recognized, attention is increasingly being paid to the role of poor interpersonal care and systemic disrespect of women as key barriers to achieving complete coverage of quality maternal health services. In addition to limiting progress towards improved maternal health outcomes, D&A is also a fundamental violation of women’s human rights and undermines the safety and effectiveness of health systems.

During recent years, disrespect and abuse has been conceptualized and categorized, therefore facilitating measurement and comparison across settings. In a global landscape analysis by Bowser and Hill, seven categories of disrespect and abuse (D&A) during childbirth emerged from qualitative and anecdotal reports: physical abuse, non-consented care, non-confidential care, non-dignified care, discrimination, abandonment, and detention in health care facilities [7].

Disrespectful and undignified care during childbirth has been documented in health facilities all over the world [7]. The underlying contributors to these forms of treatment are as complex as the health care systems that perpetuate them [8, 9]. At the interpersonal level, provider prejudices, demoralization, and burnout—all common issues experienced by overstressed healthcare workers [10]—contribute to providers delivering disrespectful care to clients [7, 11, 12]. These issues are further complicated when health facilities and health systems are overstretched, are themselves disrespectful to providers and staff, and lack accountability mechanisms and/or effective leadership [12–14].

Previous qualitative work has documented that D&A during facility-based delivery is a salient issue in Tanzania. In the Morogoro region of Tanzania, for instance, a qualitative study that included women, male partners, community health workers, and religious leaders found that all respondent groups reported personally experiencing or hearing about others’ experiences of disrespectful or abusive care during facility based childbirth [15]. In another study, women with obstetric fistula who delivered at an urban municipal hospital in Dar es Salaam recounted feeling unwelcomed by health care staff and reported experiencing abandonment as well as physical and verbal abuse during labor and delivery [16].

To date, however, there have been few quantitative estimates of the prevalence of disrespectful and abusive treatment of women during facility-based childbirth in Tanzania or elsewhere. In rural north eastern Tanzania, Kruk et al. [17] documented a prevalence of D&A ranging from 19.5 % reported upon discharge from the facility to 28.2 % reported in community-follow up interviews five to ten weeks post-delivery [17]. There have been no equivalent studies undertaken in urban regions of Tanzania, where crowded, strained facilities are particularly prevalent [18].

This article presents the findings of a study to assess the prevalence of D&A as reported by women who delivered in a large, urban referral hospital in Dar es Salaam, Tanzania, and as observed by trained individuals. This is part of the larger study that aimed to test interventions to reduce occurrence of D&A during childbirth, of which some of the findings have already been published [19].

## Methods

### Study design

This analysis presents baseline findings from an implementation research project that assessed the prevalence of disrespectful and abusive behaviors during facility-based delivery in a large, urban public hospital in Dar es Salaam, Tanzania between April and August 2013. Women were observed during labor and delivery and interviewed at two points in time: 1) immediately after delivery and preceding discharge from the facility; and 2) in their homes four to six weeks post-delivery.

### Study setting

In the Dar es Salaam region, over 90 % of the population delivers in a healthcare facility [19]. The study facility is located in the poorest district in Dar es Salaam and serves as a regional referral hospital for complicated pregnancy and maternity cases to over 70 health facilities in the district. Although the study hospital is a referral-level facility, it is common for women to bypass lower level health facilities—where they received their antenatal care—to deliver at regional hospitals. During the data collection period, more than two-thirds of respondents self-referred to the study facility for their delivery.

In Tanzania—where only 75 % of the healthcare workforce requirement is met [2]— healthcare providers often face heavy workloads in congested environments, particularly in urban areas where population density and facility-based delivery rates are high [20]. During data collection between April and August 2013, the study facility had two to three health providers in the labor and delivery ward per eight-hour shift to manage an average of 60 deliveries per day. During this timeframe, there were an average of 125 pregnancy complications, four maternal deaths, and 18 neonatal deaths per month (Facility data, 2013).

### Study sampling and data collection

The data presented here were collected through three methods: direct observations of the labor and delivery process, postpartum client interviews at the time of discharge from the facility, and community follow-up client interviews at four to six weeks post-delivery. In keeping with the Tanzanian National Institute of Medical Research (NIMR) guidelines, all pregnant women 18 years and above and admitted to the study facility for labor and delivery services during the period of data collection were eligible for this study.

All study tools were adapted with permission from a similar project conducted by the Population Council in Kenya [21]. Slight changes were made to accommodate the difference in study settings. All data collectors attended a three-day training session prior to the initiation of the study to orient them and ensure thorough understanding of the study protocol, data collection tools, and informed consent procedures. Immediately after the training, a one-day pilot of the observation and postpartum interview tools was conducted at a comparable regional referral hospital in Dar es Salaam. Written informed consent was obtained from the study facility to conduct the direct observations, which constituted consent from providers as well. Consent was sought from all eligible mothers before observation, prior to postpartum interviews, and before community follow-up interviews. Women were informed that participation was voluntary and that their participation or lack thereof would in no way impact the care they or their family members would receive now or in the future. Participants were also informed that they might end their participation at any time. The study protocol received ethical approval by the Institutional Review Board of the Tanzanian National Institute of Medical Research (NIMR) and Harvard Chan School of Public Health.

Two hundred and eight women consented for and were observed during the labor and delivery process. Eight trained nurse-midwives who were not affiliated with the study facility conducted the direct observations, each of which lasted an average of six to eight hours. When an observer finished an observation, she returned to the registration desk and approached the second woman to present at registration for participation. Consenting participants were observed from registration in the prenatal ward through the labor and postnatal wards and for up to two hours post-delivery. The study was not designed to detect D&A incidences during surgical procedures, therefore observations were stopped if the participant required a Caesarean section or experienced any other extreme complication(s) that necessitated transfer to the operating theater. Observations were conducted 24 hours a day in three shifts for 60 days. At the end of every shift, observers passed their work on to the incoming data collector to ensure complete coverage.

Trained social scientists conducted postpartum interviews with 1914 women, including 94 women who were observed (see above) and 1820 who were systematically sampled, whereby every second woman who entered the postnatal ward at the study facility was invited to participate. Consenting women were interviewed immediately before discharge from the facility—approximately three to six hours post-delivery—in a separate private room near the postnatal ward. Interviews lasted approximately 45 minutes.

Four to six weeks after delivery, researchers attempted to contact the 94 out of 197 women who were observed during labor and delivery and included in the postpartum interview sample by mobile phone for a follow-up interview. Sixty-four of these women were successfully reached and consented to a follow up interview in their home. An abbreviated version of the postpartum interview tool—in which repetitive information regarding demographics and previous care history was removed—was re-administered to the participants by the same data collectors.

### Study variables

During direct observations, data collectors reported on clinical and interpersonal aspects of care including greetings from providers to clients, infection prevention protocols, specific procedures conducted, the nature of interactions between providers and clients, and specific instances of disrespect or abuse drawn from the Bowser and Hill framework.

During the postpartum and community follow-up interviews, respondents provided information on their demographic and household characteristics, previous care history, perceived quality of care during labor and delivery, instances of disrespect and abuse the experienced and overall satisfaction with services.

The main outcomes of interest for postpartum and community follow-up interviews were client-reported experiences of each of the seven dimensions of D&A. Based on client reports, the study team divided Bowser and Hill’s category of “non-confidential care” into two distinct categories: “non-confidential care”, which was defined as violations of auditory privacy or disclosing confidential records, and “lack of privacy”, which was defined as violations of physical privacy. An error in the skip patterns resulted in questions about discrimination being asked only of women who reported experiencing physical abuse. Therefore, a representative estimate of discrimination was not obtained and is not included in this analysis.

Respondents were specifically asked about their experience of each individual category of disrespect and abuse. If a respondent reported experiencing any category of D&A, she was asked to specify the exact nature of the behavior. The specific sub-components of each category of D&A that were assessed are shown below in Table 1. Overall prevalence of D&A was calculated to include all respondents who reported any experience of any sub-type of D&A.

**Table 1:** Categories of disrespect and abuse [7]

Category of Disrespect and Abuse	Sub-components measured
Physical abuse	Kicked, pinched, slapped, episiotomy without anesthesia, pushed, raped, other
Non-dignified care	Shouted at, scolded, threatened to withhold services, laughed at or scorned, other
Non-consented care	Tubal ligation, hysterectomy, abdominal palpation, vaginal examination, episiotomy, other
Non-confidential care	HIV status shown to others, other health information shown to others, HIV status shown to non-health staff, health information discussed with non-health staff, personal issues discussed in earshot of others, other
Lack of privacy	Uncovered during delivery or examination, no screens blocking view during delivery or examination
Abandonment	While in labor, while delivering, while experiencing a complication, after delivery, other
Detention	Any

### Statistical methods

Data analysis was performed using SAS Version 9.3 (SAS Institute Inc., Cary, NC, USA). Categorical variables were summarized by proportion and continuous variables were summarized by mean and standard deviation (SD). The primary outcomes were overall disrespect and abuse (any reporting of any element of disrespect and abuse), physical abuse, non-consented care, non-confidential care, lack of privacy, abandonment and detention. To compare reports of D&A as measured during the postpartum interview and at community follow-up, χ^2^ or Fisher’s exact tests were performed. All statistical tests were two-sided with *p* < 0.05 considered significant, and all statistical analyses were carried out using the statistical software package SAS, Release 9.3 (Cary, North Carolina, USA).

## Results

Table 2 presents the socio-demographic characteristics of the postpartum and community follow-up interviews. There were no significant differences between the two samples in any of the characteristics measured. In both samples, the average age of respondents was approximately 25 years. The majority of respondents had completed a primary education, and over two-thirds in each sample were Muslim. Most respondents were married or cohabitating with a partner, and the majority in both samples was not formally employed. Approximately 90 % of respondents lived in a household headed by a male and which contained three or fewer children less than five years of age.

**Table 2:** Client demographic characteristics

Variable	Measure		*P*-value^a^
	Postpartum Interview *N* = 1914	Community Follow-Up *N* = 64	
*Socio-Demographic Characteristics*			
Age in years, Mean (SD)	25.7(5.9)	25.2(5.9)	0.52 ^b^
HIV status, n (%)			0.46^c^
*Positive*	145(8)	3(5)	
*Negative*	1725(90)	60(94)	
Education Level, n (%)			0.52 ^c^
*Primary incomplete*	32(2)	0(0)	
*Primary complete*	1176(61)	40(65)	
*Secondary+*	531(28)	22(35)	
Religion, n (%)			0.81
*Muslim*	1426(75)	49(77)	
*Christian*	417 (22)	14 (22)	
Marital Status, n (%)			0.55 ^c^
*Never Married*	281(5)	7(11)	
*Married or living together*	1605(84)	56(88)	
*Divorced/Separated/Widowed*	28((1)	1(2)	
Number of children <5 years in household, n (%)			0.34
*0 children*	1185(62)	44(69)	
*1-3 children*	643(34)	18(28)	
*>3 children*	12(1)	1(2)	
Head of Household, n (%)			0.75
*Man*	1724(90)	58(91)	
*Woman*	185(10)	6(9)	
Occupation Status, n (%)			0.17
*Employed*	795(42)	22(34)	
*Unemployed*	1119(58)	42(67)	

^a^Pearson χ^2^unless noted

^b^2-sided t-test

^c^Fisher’s exact

The delivery characteristics and service utilization histories of the postpartum and community follow-up interview samples were also not significantly different in any measured category (Table 3). Approximately one-third of each sample was nulliparous, and one-third reported having had a previous delivery at the study facility. Nearly all respondents had attended at least one antenatal care visit, with approximately 60 % of each sample reporting having attended four or more. The majority of respondents came directly to the study facility for the recorded delivery without a referral. Nurses and midwives conducted the majority of deliveries in both samples.

**Table 3:** Service utilization history and delivery characteristics

Variable	Measure		*P*-value^a^
	Postpartum Interview *N* = 1914	Community Follow-Up *N* = 64	
*Delivery characteristics*			
Number of previous births, n (%)			0.82^b^
*0*	600 (31)	19(30)	
*1-2*	875(46)	30(47)	
*3-4*	311(16)	11(17)	
*5+*	79(4)	1(2)	
Antenatal Care visits made, n (%)			0.79^b^
*No visit*	35(2)	0(0)	
*1 visit*	45(2)	1(2)	
*2 visits*	148(8)	5(8)	
*3 visits*	520(27)	22(34)	
*4+ visits*	1152(60)	36(56)	
Ever had a previous delivery at study facility, n (%)			0.72
*Yes*	661(35)	25 (39)	
*No*	930(49)	29(45)	
*Don’t Know*	314(16)	10(16)	
Referrals for current delivery, n (%)			0.69 ^b^
*Came directly to study facility*	1488(78)	52(81)	
*Sent from dispensary to study facility*	251(13)	7(11)	
*Sent from health centre to study facility*	94(5)	4(6)	
*Other transfer*	25(1)	1(2)	
Type of Provider Conducted Delivery, n (%)			0.66^b^
*Doctor/Clinical officer/Intern*	181(9)	7(11)	
*Nurse/midwife*	1718(90)	56(88)	
*Provider unknown*	12(1)	1(2)	
*No one*	2(0.1)	0(0)	
*Birth before arrival at facility*	1(0.1)	0(0)	
Time of delivery, n (%)			0.79
*Day*	1048(55)	34(53)	
*Night*	865(45)	30(47)	

^a^Pearson χ^2^unless noted

^b^Fisher’s exact

Table 4 presents the self-reports of disrespect and abuse stated during the postpartum and community follow-up interviews. During postpartum interviews, 15 % of respondents reported experiencing any D&A. Reporting of D&A was significantly higher (*P* < 0.001) during community follow-up interviews, in which 70 % of respondents reported any experience of D&A. A significantly higher proportion of respondents reported experiencing physical abuse (52 % vs. 5 %, *P* < 0.001), non-consented care (5 % vs. 0.2 %, *P* < 0.001), non-confidential care (54 % vs. 2 %, *P* < 0.001), lack of privacy (53 % vs. 2 %, *P* < 0.001), non-dignified care (53 % vs. 6 %, *P* < 0.001), and abandonment (52 % vs. 8 %, *P* < 0.001) during community follow-up interviews compared to postpartum interviews, respectively.

**Table 4:** Client reports of disrespect and abuse

Type of Disrespect and Abuse	Postpartum Interview *N* = 1914 n (%)	Community Follow-Up *N* = 64 n (%)	*p*-value
Any form of disrespect or abuse	278(15)	50(70)	<0.001
Physical abuse	84(5)	33 (52)	<0.001
*Kicked*	2(0.1)	1(1)	0.03
*Pinched*	22(1)	3(5)	0.02
*Slapped*	23(1)	17(27)	<0.001
*Pushed*	12(0.6)	4(6)	<0.001
*Beaten*	4(0.2)	5(8)	<0.001
*Episiotomy without anesthesia*	1(0.1)	1(2)	<0.001
*Tied to the delivery bed*	2(0.1)	0 (0)	0.80
*Other*	17(0.9)	9(14)	<0.001
Non-consented care	4(0.2)	3(5)	<0.001
*Abdominal Palpation*	2(0.1)	0 (0)	0.79
*Vaginal Examination*	4(0.2)	1(2)	0.35
*Episiotomy*	0(0)	1(1)	<0.001
*Other*	0 (0)	1(2)	<0.001
Non-confidential care	32(2)	34 (54)	<0.001
*Discussed personal issues in earshot of other clients*	1(0.1)	1(2)	0.004
*Health information discussed with non-health staff*	0(0)	2(3)	<0.001
*Other*	0(0)	0(0)	0.79
Lack of Privacy	35(2)	34(53)	<0.001
*No screens blocking view during delivery or examination*	16(0.8)	29(45)	<0.001
*Uncovered during delivery or examination*	17(0.9)	31(48)	<0.001
Non-dignified care	121(6)	34(53)	<0.001
*Shouted at*	35(2)	24(38)	<0.001
*Scolded*	90(5)	16(25)	<0.001
*Threatened to withhold services*	1(0.1)	1(2)	0.04
*Called by insulting name*	3(0.2)	0(0)	0.71
*Laughed at or scorned*	3(0.2)	0(0)	0.71
*Other*	12(0.6)	1(2)	0.77
Abandonment of care	147(8)	33(52)	<0.001
*While in Labor*	104(5)	22(34)	<0.001
*While Delivering*	60(3)	12(19)	<0.001
*While experiencing a complication*	1(0.1)	0(0)	0.79
*After delivery*	2(0.1)	0(0)	0.71
*Other*	5(0.3)	0(0)	0.64
Detention in facilities	4(0.2)	1(2)	0.17

In addition to the categories of D&A presented in Table 4 and included in calculations of “overall D&A”, responses given by women indicated that disrespect and abuse were evident in ways not explicitly included in the Bowser and Hill framework. Table 5 presents information on the prevalence of two additional categories of behaviors that may be considered disrespectful or abusive and which were prevalent in the study facility. Lack of information was frequently reported by respondents and was separated from non-consented care. This differentiation was considered important by many local stakeholders, who believe that violating consent (e.g. performing a procedure when the woman was not asked for permission) was an intentional act of D&A, while lack of information was passive (e.g. the provider may neglect to mention information due to time constraints). Additionally, respondents were asked to report whether, at any time during their stay at the study facility, they felt or perceived that they were asked for favors or money other than official costs of service. Reports between the two surveys found 5 % and 14 % of women in the postpartum and community follow-up surveys reporting being asked for informal fees, respectively. The difference was not statistically significant.

**Table 5:** Other forms of D&A reported by clients

	Postpartum Interview n (%)	Community Follow-Up n (%)	*p*-value
Lack of Information (*N* = 1799 and 69, respectively)			
*No information given on ward environment (e.g. where the bathrooms are)*	1323(69)	50(78)	0.87
*No information given on meal times and what to eat or not eat*	1886(99)	64(100)	0.22
*Findings of general examination not explained*	1728(90)	57(89)	0.041
*Findings of vaginal examination not explained*	1297(68)	47(73)	0.019
*No information given on progress of labor*	1555(81)	53(83)	0.09
*No information given on movement during labor*	1856(97)	63(98)	0.34
*No information given on when to breastfeed baby*	1772(93)	63(98)	0.04
Asked for informal fees (*N* = 860 and 70, respectively)	103(5)	9(14)	0.55

In addition to the categories shown in Tables 4 and 5, 6 % of respondents in the postpartum interview and 59 % in the community follow-up interview reported experiencing some other form of D&A that they had not yet reported as a particular category of D&A. The open-ended responses provided by respondents show the complexity of reporting such behaviors and the interconnectedness of the categories presented.

**Fig. 1:**
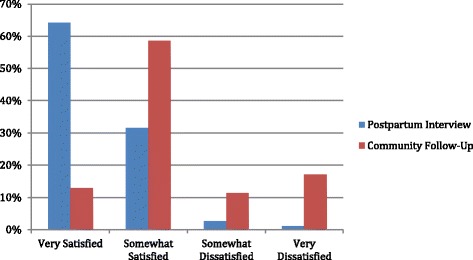
Client satisfaction with overall experience during delivery

**Fig. 2:**
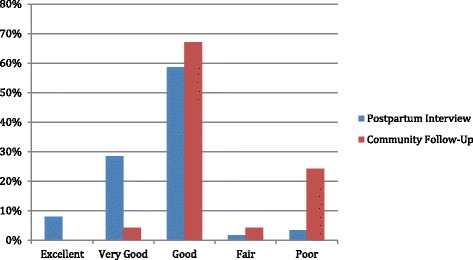
Client ratings of the respect shown to them by providers during delivery

During postpartum and community follow-up interviews, respondents were also asked about their satisfaction with their delivery experience. As shown in Fig. 1, reports of satisfaction were much higher during postpartum interviews than community follow-up interviews, and the distribution of answers between the two surveys is significantly different (*P* < 0.001). During the postpartum interview, only 1.2 % of respondents reported being somewhat or very dissatisfied; this percentage jumped substantially during community follow-up to 28.6 %. Respondents were also asked to rate the respectfulness of providers during their labor and delivery (Fig. 2). Again, reports at community follow-up were significantly less positive than the postpartum interview, with 24.3 % of respondents in the community follow-up interview reporting “poor” provider respect compared to 3.3 % during the postpartum interview (*P* < 0.001).

The direct observation checklist used for this study did not include questions that were expansive enough to capture all forms of disrespect and abuse that occurred. Therefore, we are not reporting an overall observed prevalence of D&A. However, a subset of specific events that were recorded by observers and can be compared to respondents’ self reports are shown in Table 6. Direct observations of client-provider interactions during labor and delivery documented a high incidence of disrespectful and abusive behaviors. For example, over 84 % of women were not asked for consent for examinations they received in the antenatal ward (non-consented care) and one-fifth of women had their confidentiality violated during history taking in the antenatal ward. Lack of privacy was particularly prevalent, with approximately 58 % of women not being covered during delivery and over 84 % of women required to share a bed in the postnatal ward. Furthermore, two-thirds of women who were observed were put in beds in the postnatal ward that were not clean. Open-ended responses recorded by observers provide additional examples of the types of disrespectful and abusive behaviors occurring in the study facility:

*“The nurse shouted and spoke ridiculing words while the mother lay there naked and uncovered.” (Direct Observation)*

*“She delivered on the floor as the nurse was still shouting, she continued to shout at her and she didn’t even look to see how the baby came out.” (Direct Observation)*

**Table 6:** Observed disrespect and abuse

Type of disrespect and abuse observed	Incidence *N* = 197 n (%)
Physical abuse	
*Episiotomy performed without anesthesia given (n = 17)*	9(5)
Non-consented care	
*Lack of consent for first examination in antenatal ward*	166(84)
*Lack of consent for vaginal examination in antenatal ward*	160(81)
Non-confidential care	
*Mother’s history taking findings shared when others could hear*	37(19)
Lack of privacy	
*No partitions separating beds in antenatal ward*	10(5)
*Partitions do not give privacy in antenatal ward*	50(25)
*Mother not covered during examination in antenatal ward*	46(23)
*Mother not covered while being moved from antenatal ward to delivery room*	27(14)
*Mother not covered during delivery*	115(58)
*Partitions not closed during delivery*	53(27)
*Mother not well covered after third stage of labor*	23(12)
*Mother not given a bed to herself in post-natal ward*	166(84)
*No partitions/curtains between beds in post-natal ward*	167(85)
*No partition/curtain during post-natal examination, if done (n = 19)*	9(50)
*Mother not covered during post-natal examination, if done (n = 19)*	9(50)
Non-dignified care	
*Mother not welcomed in a kind and gentle manner*	50(26)
*Use of non-dignified language during history taking*	9(5)
*Use of harsh tone or shouting during history taking*	13(62)
*Bed in post-natal ward not clean*	122(67)
*Bed in post-natal ward not covered with a bed sheet*	180(91)

## Discussion

The findings presented here are among some of the first quantitative measures of disrespect and abuse during facility-based childbirth, and the first known findings that are specific to an urban hospital environment. Overall, 15 % of respondents reported experiencing some form of disrespectful or abusive behavior when interviewed three to six hours postpartum. This number rose dramatically to 70 % when respondents were interviewed weeks later in their homes. The most commonly reported categories of D&A in the postpartum interview were abandonment, non-dignified care, and physical abuse. During community follow-up interviews, more than 50 % of women reported experiencing D&A for nearly all categories, with only non-consented care and detention being reported by less than half of respondents.

The difference in reporting of D&A between the two time periods is striking. Similarly, levels of satisfaction with overall care experience and perceptions of provider respectfulness were significantly lower in the community follow-up than postpartum interviews. Thus, reports of both experienced and perceived elements of care were significantly different between the two interviews and both were significantly more negative at community follow-up. The difference cannot be explained by demographic characteristic or reproductive care history profiles of respondents, because women in both groups were very similar. Although the sample size of the community follow-up is substantially smaller, it was randomly selected. We therefore can safely hypothesize that the full sample of postpartum interview respondents (*n* = 2000) would have responded similarly had they been interviewed at community follow-up.

We hypothesize that these differences may be due in part to the fact that women immediately postpartum can be overwhelmed by feelings of exhaustion and relief [22] and may not have time to reflect on their experience until much later. Despite study staff assurances that responses would not be linked to the women or affect their future care in any way, it is also possible that these differences may be influenced by courtesy bias or women not feeling comfortable reporting a negative experience while still at the facility. Other studies, however, have shown that reporting of other subjective indicators—such as staff friendliness or satisfaction—can be much higher during facility-based than household-based interviews [23]. It is also possible that the postpartum interview sensitized respondents to the issue of disrespect and abuse, making them more likely to consider the issue during community follow-up interviews. More research is needed to determine why women’s perceptions of their childbirth experience change over time and in different locations and circumstances.

Disrespect and abuse is a complex phenomenon to measure. In an attempt to increase the validity of our results, we triangulated data sources on the observed incidence of disrespectful and abusive behaviors during client-provider interactions during childbirth and the incidence reported by women in the immediate and late postpartum periods. Data obtained through each of these methods have specific limitations. Not all constructs of D&A can be captured from an outside perspective; for example, humiliation through non-dignified care is subjective to the feelings of the woman. However it is clear from the data presented that external observers recorded an extremely high incidence of some forms of D&A. Over 84 % of women observed had to share a bed in the post-natal ward, a position that was likely to compromise their privacy, confidentiality, and dignity. These discrepancies between women’s self-reports of disrespect and abuse—both during postpartum and community follow-up interviews—and the incidence recorded by direct observers indicate that at least some forms of disrespect and abuse have been normalized by a large segment of the patient population.

The forms of disrespect and abuse reported in this study comprise two broad, non-mutually exclusive categories: those that are inherently interpersonal in nature and those that are likely driven or perpetuated by facility and health system shortages and failures. Between April and August 2013 when data were being collected, a total of 8869 deliveries took place in the study facility for an average of 60 deliveries per day. Additionally, 624 pregnancy complications were recorded, as were 20 maternal deaths and 90 neonatal deaths. Despite this high patient load and poor maternal health outcomes, the study facility was dramatically understaffed. During the study period, there were only three providers per eight-hour shift in the labor and delivery ward, a maximum of six providers per shift in the antenatal ward, and a maximum of three providers per shift in the postnatal ward. These conditions—which are increasingly common in urban areas throughout resource-limited countries as facility-based delivery rates increase [4, 11]—are in and of themselves disrespectful to both the staff and the women who experience them. Infrastructural and staffing shortages and deficiencies likely play a substantial causal role in the high rates of privacy/confidentiality breaches and abandonment of care.

However, the reports of disrespect and abuse presented here cannot be solely attributed to such institutional and systemic issues.

Many instances of disrespectful behavior have obvious implications for maternal and newborn safety, such as the accounts above of women delivering alone because a provider refused to help. However, even categories of disrespect and abuse that may be considered to be “lesser” offenses or “lower risk”—such as scolding, humiliation, or shouting—undermine patient-provider trust and can have insidious effects on the safety and culture of a health care facility [12]. For example, it is highly likely that a mother who has been ignored or shouted at throughout the course of her labor would hesitate to inform a health care provider of danger signs for fear of further maltreatment.

Taken together, these narratives show that despite taxing conditions, disrespect and abuse are never inevitable. There is a clear need to improve the conditions of care and to better understand providers’ perceptions and experiences to ensure that their needs are being met, and that they are supported and encouraged to act in a kind and caring manner.

### Study limitations

The study tools were developed and validated for a research program that measured disrespect and abuse in Kenya, but were not validated in Tanzania. Similarly, the checklist employed for direct observations of client-provider interactions during labor and delivery was not originally developed for measurement of disrespect and abuse, and therefore did not holistically measure all manifestations of these behaviors. Finally, budget and time constraints prevented investigators from obtaining a statistically significant sample of community follow-up interviews, which would have allowed for more precision and reliability of these data. In order to address this gap we have used the variety of methods to allow for triangulation between perspectives of disrespectful and abusive events to provide a more holistic understanding of the scope and magnitude of these issues.

## Conclusion

This study is an illustrative example of why using facility-based delivery rates as a proxy indicator for quality maternal health care is not appropriate. The Dar es Salaam region has a 90 % facility-based delivery rate, yet maternal health outcomes in the study facility and region are shockingly poor. In many urban areas throughout the resource-limited countries, improved and incentivized access to services has led to an increase in women seeking facility-based care during childbirth. As a result, many urban hospitals such as the study facility have extremely high patient flow and yet are faced with significant resource and staff shortages, which is likely to be one of the key drivers of disrespect and abuse. When matters of quality and acceptability are not addressed in these settings, situations arise in which health care providers work in demoralizing and stressful conditions and a significant proportion of women are unsatisfied with their care and suffer a violation of their basic human rights. In summary, disrespect and abuse is an indicator of a health system in crisis [17]. Additional research and programming is needed to strengthen health systems to provide acceptable quality care—with the principles of respectful and dignified maternity care at the core of service delivery—in order to realize every woman’s right to respectful care and to improve maternal health outcomes.

## Abbreviations

D&A: Disrespect and Abuse
MDG: Millennium Development Goal
NIMR: National Institute of Medical Research
SD: Standard Deviation
